# First attempt to motion corrected flow encoding using free-breathing phase-contrast CINE MRI

**DOI:** 10.1186/1532-429X-14-S1-W53

**Published:** 2012-02-01

**Authors:** Christophe Meyer, Pierre-Andre Vuissoz, Damien Mandry, Jacques Felblinger

**Affiliations:** 1 IADI / INSERM U947, Vandoeuvre-les-Nancy, France; 2 CHU Nancy, Vandoeuvre-les-Nancy, France; 3 Université de Lorraine, Nancy, France; 4 CIC-IT Nancy, Nancy, France

## Summary

This study demonstrates the feasibility of free-breathing phase-contrast CINE MRI without averaging. A new version of the CINE GRICS algorithm[1] was used to correct for motion.

## Background

Phase-contrast MRI encodes speed and direction of moving spins by means of toggling a bipolar gradient. It is a valuable tool for assessing conditions affecting the vascular system by measuring the velocity of flowing blood[2]. Clinically, this sequence is performed in breath-hold or in free breathing but, in the latter case, using signal averaging. We propose to demonstrate the feasibility of free-breathing phase-contrast CINE MRI without averaging exploiting the acquisition redundancy by applying a new version of the CINE GRICS algorithm[1] to correct for motion.

## Methods

Cardiac examination (approved by our local ethics committee) was performed on one normal volunteer during which three 2D phase-contrast CINE MRI sequences (common parameters: 256x128 acquisition matrix, 6 views per segment (vps), 32 reconstructed cardiac phases, 150 cm/sec VENC, slice direction velocity encoding, 5 mm slice thickness, 44 cm FOV, 62.5 kHz bandwidth, 3.05/8.08 ms TE/TR, 15° flip angle) were acquired on a 3T scanner (Signa HDxt, GE Healthcare, Milwaukee, WI) with a 8-element cardiac coil : (1) breath-held (2) averaged (3 NEX) in free breathing (3) in free breathing storing the raw data of 3 NEX to an external computer for offline processing. Signals from a respiratory belt were carried by a custom Maglife patient monitoring system (Schiller Medical, France) and recorded with a dedicated home-made hardware.

**Figure 1:**
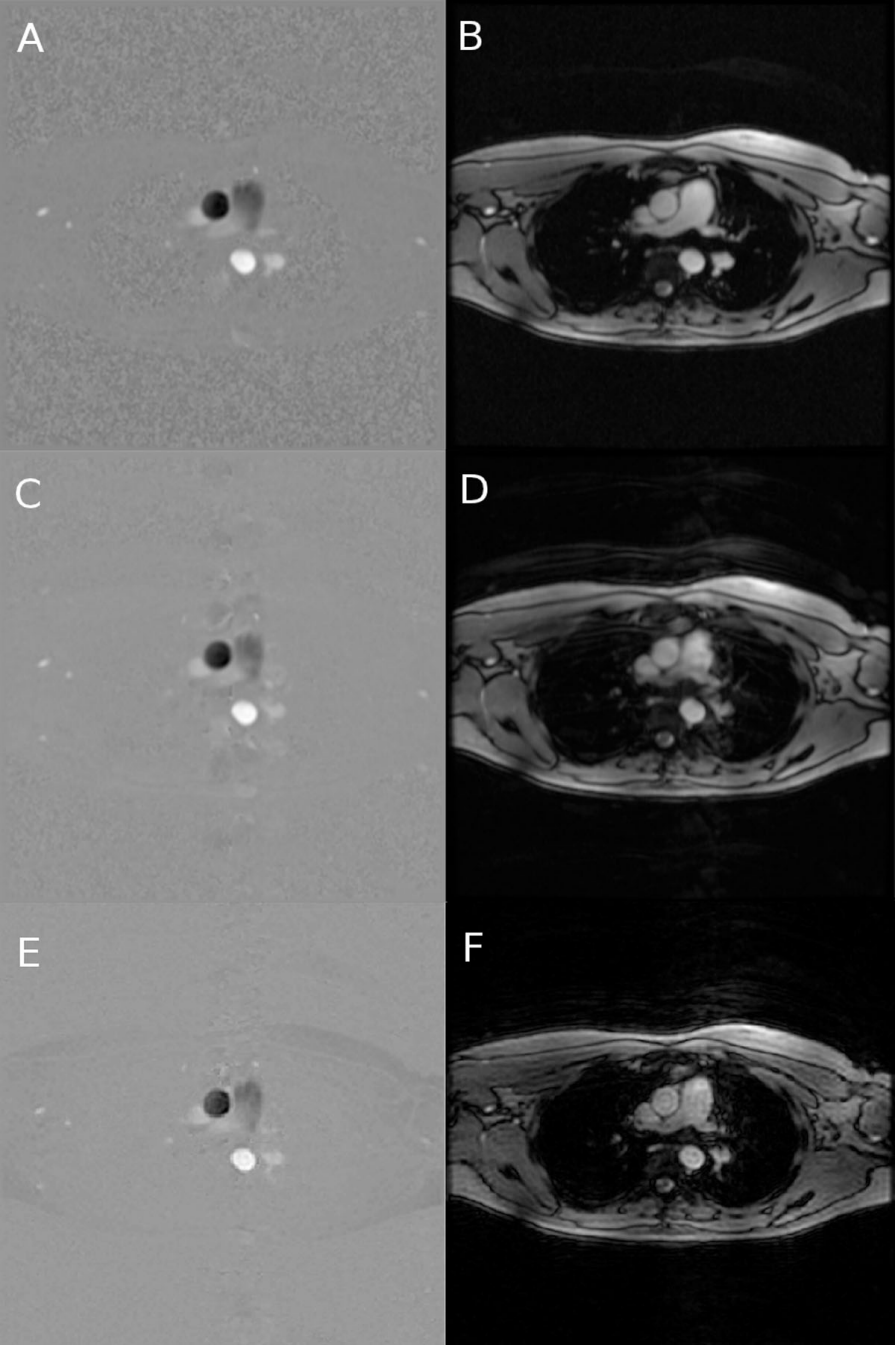
Section of the aortic arch at systolic peak velocity. Phase difference images (A,C,E) and magnitude images (B,D,F) of phase contrast CINE loops, from top to bottom : apnea, average and PC CINE GRICS.

**Figure 2:**
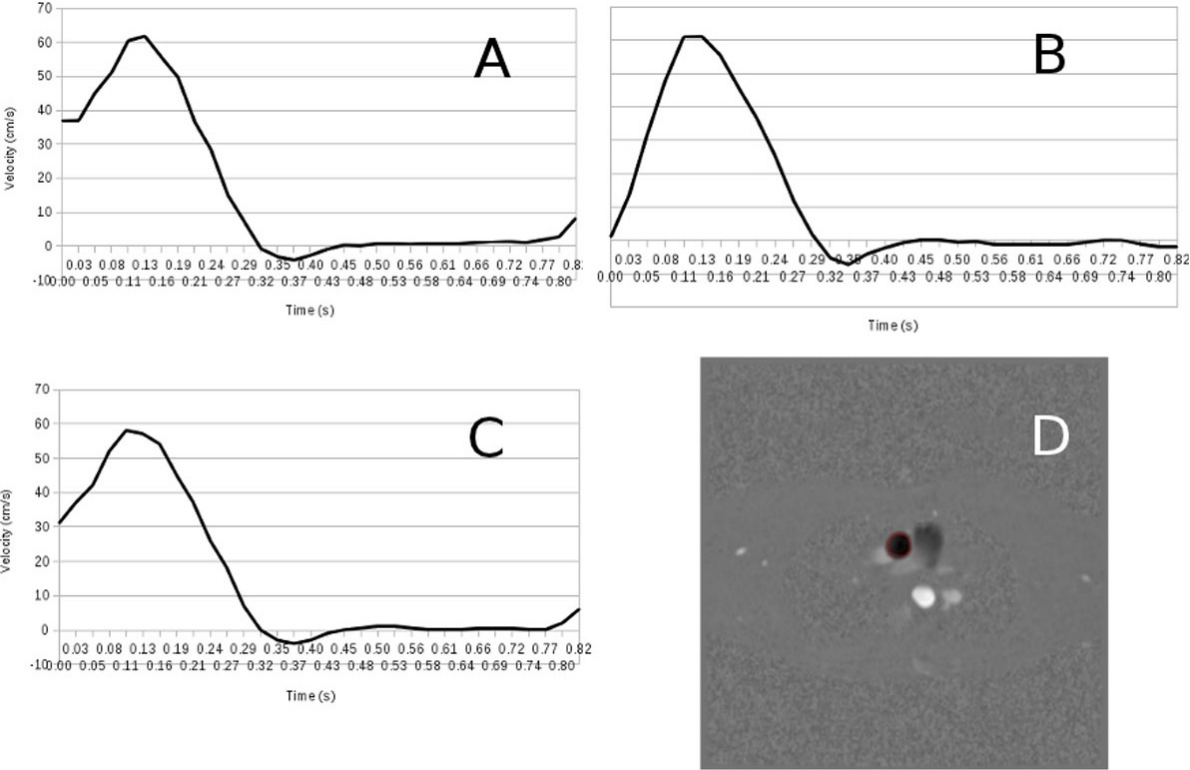
Velocity curves in an ascending aorta ROI (D) from phase contrast CINE scans in breath-hold (A) in free breathing with averaging (C) and in free breathing with PC CINE GRICS (B).

Offline processing consisted of splitting the raw data from the 2 velocity encoding steps thus giving 2 sets of raw data. They were formatted and processed separately, along with the respiratory signals, by the CINE GRICS algorithm on a 16-node compute cluster. Total reconstruction delay was 15 min. Phase difference images (Figure 1) were created. Images were analysed by an experimented radiologist using CV Flow (GE Medical Systems) and velocity curves were produced (Figure 2) for the ascending aorta ROI.

## Results

Figure 1 shows a section of the aortic arch from the 3 sequences (all same plane coordinates and cardiac phase). Averaged acquisition exhibits blurring on the images generated by the manufacturer’s software. Motion correction using GRICS removes almost all ghosting artefacts and improves vessel contrast delineation. Figure 2 shows that velocity curves from the breath-held, free breathing and PC CINE GRICS scans present the same features (note that curves are shifted due to trigger delay implementations).

## Conclusions

We have demonstrated that motion corrected free-breathing phase-contrast CINE MRI using GRICS is feasible. Future work will focus on (1) a 3D PC CINE implementation and (2) increased resolution for sharp, small structures imaging.
